# Treasures from trash in cancer research

**DOI:** 10.18632/oncotarget.28308

**Published:** 2022-11-17

**Authors:** Fabiano Cordeiro Moreira, Dionison Pereira Sarquis, Jorge Estefano Santana de Souza, Daniel de Souza Avelar, Taíssa Maria Thomaz Araújo, André Salim Khayat, Sidney Emanuel Batista dos Santos, Paulo Pimentel de Assumpção

**Affiliations:** ^1^Núcleo de Pesquisas em Oncologia/Universidade Federal do Pará, Belém, Pará, Brazil; ^2^Instituto Metrópole Digital/Universidade Federal do Rio Grande do Norte, Natal, Brazil; ^3^Instituto de Ciências Biológicas/Universidade Federal do Pará, Belém, Pará, Brazil; ^*^Co-first authors

**Keywords:** cancer metagenomics, cancer sncRNA expression, RNA-Seq variant calling

## Abstract

Introduction: Cancer research has significantly improved in recent years, primarily due to next-generation sequencing (NGS) technology. Consequently, an enormous amount of genomic and transcriptomic data has been generated. In most cases, the data needed for research goals are used, and unwanted reads are discarded. However, these eliminated data contain relevant information. Aiming to test this hypothesis, genomic and transcriptomic data were acquired from public datasets.

Materials and Methods: Metagenomic tools were used to explore genomic cancer data; additional annotations were used to explore differentially expressed ncRNAs from miRNA experiments, and variants in adjacent to tumor samples from RNA-seq experiments were also investigated.

Results: In all analyses, new data were obtained: from DNA-seq data, microbiome taxonomies were characterized with a similar performance of dedicated metagenomic research; from miRNA-seq data, additional differentially expressed sncRNAs were found; and in tumor and adjacent to tumor tissue data, somatic variants were found.

Conclusions: These findings indicate that unexplored data from NGS experiments could help elucidate carcinogenesis and discover putative biomarkers with clinical applications. Further investigations should be considered for experimental design, providing opportunities to optimize data, saving time and resources while granting access to multiple genomic perspectives from the same sample and experimental run.

## INTRODUCTION

Advances in molecular biology and bioinformatics allow for unprecedented data generation, thereby promoting a greater understanding of many physiological and pathological phenomena in human organisms [1]. Nevertheless, the amount of data produced in each of these experiments usually surpasses the main focus of the proposed investigations [2, 3]. Currently, the strategies for finding molecular markers in cancer research have accumulated an enormous amount of unintended information. Most of these supposed useless data are often treated as trash and remain unexplored. However, in some cases, treasures hidden in these data are discarded [2, 4]. Here, we demonstrate potential strategies to benefit from nontargeted information resulting from high-throughput cancer investigations.

### Human cancer genome and metagenomics

The last three decades have been extremely prolific regarding the generation of cancer genome data [5, 6]. Thousands of cancer genomes have been sequenced and analyzed around the world, and millions of dollars have been invested in such a “gold rush”, thus leading to an immense improvement in our understanding of disease [7, 8]. After sequencing, bioinformatics teams deeply explored the data according to the investigation goals. This difficult task of processing human genome data remains ongoing, especially in case of additional questions emerging after initial investigations. Nevertheless, a fraction of data is uncharted.

Recently, a new gold rush was launched: metagenomics. Again, large investments have been made toward efforts to discover the role of the microbiome in many cancer types [9–11]. Moreover, data generation is followed by bioinformatics, which has the task of interpreting the data and generating new hypotheses. New horizons in cancer knowledge are arising and on their way to be implemented in clinical practice.

These two gold rushes have several common features: most experiments collect fresh samples from human tumors and paired noncancer tissues; sequencing procedures result in a large and complex amount of data; bioinformatics teams select the desired information; and nonessential information (“trash”) is discarded, aiming at focusing on the research goals and reducing confounding data [12–15]. Next-generation sequencing (NGS) data are available in public data banks, such as GEO (http://www.ncbi.nlm.nih.gov/geo) [16], and they usually provide access to raw genomic data, thereby increasing the credibility, transparency, and reproducibility of the results, while also allowing for additional investigations.

The steps of both cancer genomics and metagenomics experiments are similar. Both investigations are usually based on the acquisition of fresh samples from tumors followed by DNA extraction, library preparation and sequencing. In fact, human and nonhuman DNA are available at this point of the experiment. Nevertheless, according to the investigator, focus is driven to either human DNA for genomics or nonhuman DNA for metagenomics. The unexplored data from each case might harbor some of the objectives of other studies, paving the way for an integrative exploration of both human and nonhuman information.

Therefore, NGS genomic data have sufficient information for taxonomic investigation with similar precision of dedicated metagenomic experiments. In an attempt to test this hypothesis, we performed additional analyses using publicly available gastric, prostate and bladder cancer genomic data to explore metagenomic information.

### Exploring additional small noncoding RNAs from miRNA sequencing

Total RNA-seq analyses are NGS experiments that have the most significant opportunity to explore new data. The majority of these experiments focus only on specific types of transcripts, such as mRNA, lncRNA, or miRNA. However, this strategy allows for exploration of several RNA molecules simultaneously. Additionally, from total RNA-seq data, it is possible to detect new genomic variants, providing potentially insights to such experiments. Furthermore, nonhuman microbiome expression data (metatranscriptome) are captured from total RNA-seq. Since both human and nonhuman data are usually sequenced, the identification of microbiome gene expression and several downstream investigations are possible, such as taxonomic profiles and host-microbiome interactions [17].

Most small noncoding RNA (sncRNA) investigations in cancer research address miRNA expression as potential cancer biomarkers and targets for therapy [18–20]. Usually, miRNA sequencing pipelines identify and select small RNA fragments and subsequently quantify each known miRNA [21, 22]. However, among these small fragments, many sequences are not representative of miRNAs and are excluded from analyses as contaminants. Some of these excluded sequences correspond to other classes of noncoding RNAs that increase the cancer process, as is the case for piwi-interacting RNAs (piRNAs) [23, 24]. An integrative strategy should include other noncoding RNAs (ncRNAs), thus harnessing the full potential of all samples and laboratory work, while opening new possibilities for the discovery of regulatory networks.

We hypothesize that evaluating the discarded sequences from miRNA sequencing data enables the identification of sncRNAs with potential value as cancer biomarkers or treatment approaches. To test this hypothesis, gastric, bladder and prostate cancer miRNA-seq data were explored.

### Hidden markers in adjacent to tumor samples

Most gene expression investigations in solid tumors rely on comparisons between tumors and adjacent to tumor samples, considering adjacent samples as normal controls. Nevertheless, such specimens harbor molecular alterations that are insufficient to cause cancer but differ from normal tissues collected from noncancer patients [21, 25, 26]. The focus of such experiments is to identify differentially expressed genes between cancer and adjacent to cancer samples because these genes shed light on the molecular events involved in the carcinogenic process [27, 28]. However, initial molecular events are likely present in both adjacent to tumor and tumor samples; thus, searching for differences between them may not reveal these important carcinogenic molecular events [29].

In some cases, however, the analyzed transcripts contain neglected information, such as concomitant expression of oncogenes and expression of mutated genes from both adjacent to tumor and cancer tissues. Once more, these relevant data are likely to be discarded because the goal is generally to identify differential expression and not concurrent variant patterns [27].

Thus, adjacent tumor tissue, which is often used only as a gene expression control, potentially has somatic alterations common to cancer that may hold an essential role in understanding the first steps of carcinogenesis. Aiming to prove this concept, we explored sequencing data from paired gastric tumors and adjacent to tumor tissues.

## MATERIALS AND METHODS

### Data acquisition

All data were downloaded from SRA databank (https://www.ncbi.nlm.nih.gov/sra) [30]. For metagenome from genome data analysis, we employed three different cancer types: bladder cancer (PRJNA185252, 44 samples) [31], gastric cancer (PRJNA173904, 19 samples) [32], and prostate cancer (PRJNA412953, 15 samples; PRJEB6530, 20 samples) [33, 34]. For identification of additional sncRNAs from miRNA sequencing, we explored the same cancer types distributed as follows: bladder cancer (five cancer and five noncancer samples) [35]; peripheral blood of prostate cancer (32 cancer and 13 noncancer patients) [36]; and gastric cancer (eight cancer and eight noncancer samples) [20]. For variant calling from RNA-seq, we obtained data from 80 samples, which were generated from 20 gastric cancer patients and distributed as follows: 20 exome tumor samples, 20 exome blood samples, and 20 RNA-seq paired tumor and adjacent to tumor samples (Supplementary Table 1) [37].

### Quality control

All downloaded samples were analyzed using FastQC (version 0.11.2) [38]. Trimming and filtering were performed using Trimmomatic (version 0.36) [39]. The parameters for each analysis were chosen based on the visual evaluation performed with FastQC, quality of data, data origin (sncRNA, RNA, or DNA), and type of sequencing (paired-end or single-end). The parameters and quality values (QVs) for each analysis are described in Supplementary Table 2.

### Read alignment

To analyze metagenomic data from genomic sequencing, we used Centrifuge Aligner software (version 1.0.4-beta) [40], mapping reads to bacteria, archaea, viruses, and human sequences.

STAR aligner (version 2.7.0) [41] was used to map reads and identify additional sncRNAs and perform variant calling from RNA-seq data analysis. The genome version used in both analyses was HG19 (version 37.7; http://www.ensembl.org/info/data/ftp/). Since some sncRNAs may be repeated in several sites in human genome, the aligner parameters were adjusted, allowing at least 100 repetitions in genome. To improve identification of other sncRNAs, such as piRNAs in case multimapping occurred, the best alignment score was selected.

Exome samples were also mapped to HG19 human reference genome (version 37.7; http://www.ensembl.org/info/data/ftp/) with Burrow Wheeler Aligner (BWA; version 0.7.15) [42] using default parameters.

### sncRNA expression data

For each sample, three annotations were performed for transcript quantification using htseq-count software (version 0.6) [43]. First, mirBase annotation [44] was used to quantify microRNA expression. Reads identified as miRNAs were quantified and filtered out from the .sam file. Next, piRbase annotation [45] was used to perform piRNA expression quantification, and reads identified as piRNAs were also quantified and filtered out from the .sam file. The remaining .sam file was then used for the identification and quantification of other transcripts with ENSEMBL annotation (https://www.ensembl.org). Since there are several overlapping sequences in piRbase, we used BEDtools (version 2.17) [46] to merge overlapping sequences into unique sequences, thus avoiding ambiguous recognition.

### Taxonomic identification

Taxonomic identification consisted of identifying reads that did not align to the human genome but displayed a minimum alignment score of 60%. The results were reclassified with Recentrifuge (version 1.0.3-beta) [47]. This tool increases identification precision by using two approaches. One that considers a minimum score (here considered as 50) and a robust algorithm to remove contaminants. We employed genus-level taxonomic classification for comparison to literature data.

### Variant calling from RNA-seq and DNA-seq data

Duplicated reads were removed using Picard Tools (version 2.18; http://broadinstitute.github.io/picard). The Genome Analysis Toolkit (GATK version 4.1.2) [48] was used for local realignment and recalibration.

All variants were called using BCFtools (version 1.8) [49] on exonic regions. Low coverage variants were filtered out (less than five variant reads or variant read depth less than 20% of total depth). We called somatic variant filtering out blood variants from tumor and adjacent to tumor samples. BCFtools was applied to compare and identify common variants between tumor and adjacent to tumor samples, and Ensembl Variant Effect Predictor (VEP; https://www.ensembl.org/Homo_sapiens/Tools/VEP) [50] was used for common variant annotation.

### Statistical and graphical analyses

To compare taxonomic profiles, we filtered all data for the top 40 most relatively abundant genera (representing >90% of reads in all cancer types; Supplementary Table 3). We also converted the data to presence/absence to avoid sequencing biases and compared taxonomic profiles at genus level. The hypergeometric distribution was used to test significant overlap among microorganisms identified by the proposed whole-genome sequencing captured data (WGScd) and other literature data.

*P* values were adjusted for multiple testing using Benjamini-Hochberg false discovery rate (FDR) adjustments [51]. Alpha diversity was calculated using Simpson’s diversity index with Vegan library [52], which was implemented in R, and significant differences between the WGScd and literature data were identified using the Kruskal–Wallis test followed by Dunn’s post hoc test. The Vegan library was also employed to calculate and plot rarefaction curves.

To identify additional differentially expressed (DE) sncRNAs not addressed by the original authors, we used DESeq2 package [53], which was implemented in R, to analyze gastric, bladder, and prostate cancer experiments. SncRNAs satisfying the following criteria were tagged as differentially expressed: |log2(fold-change) | >1 and *p* value < 0.05. All graphics were created in the R statistical platform using the Venn [54] and ggplot2 [55] packages. The R codes for all statistical analyses and plots are provided in the Supplementary Material.

## RESULTS

### Metagenomic analysis from genomic data

Three different cancer types from bladder, prostate, and gastric tumors were explored to obtain additional findings. This methodology is hereafter referred to as “Whole Genome Sequencing captured data” (WGScd) to differentiate it from other metagenomic analyses. The results were compared to metagenomic literature data.

**Figure 1:**
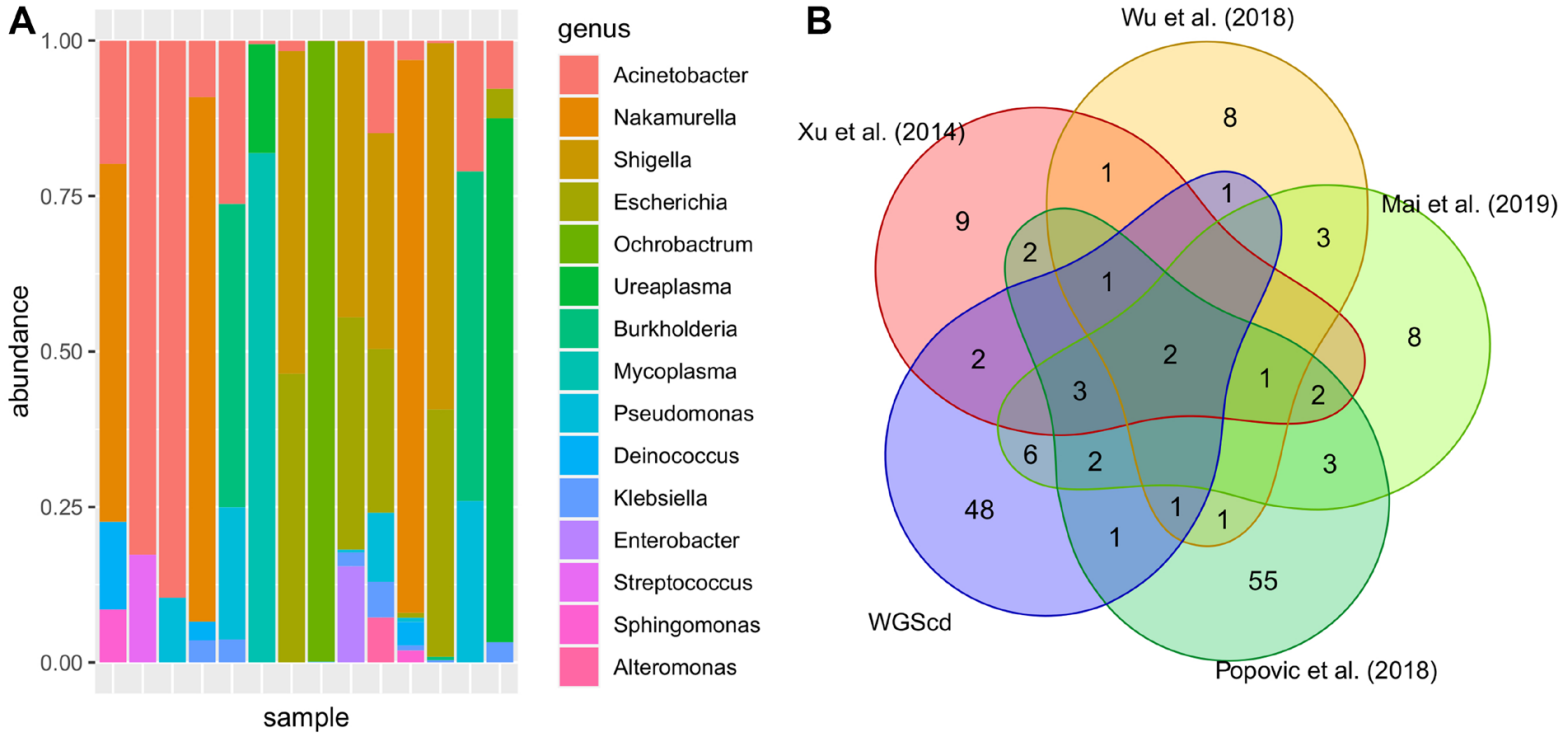
Most abundant bacteria taxa found in bladder analysis. In (**A**), relative genus abundance among samples. In (**B**), presence/absence Venn diagram: Bladder cancer tissue metagenomic profile obtained from Whole Genomic Sequencing captured data (WGScd) compared with literature research of urine bladder cancer metagenomic profile obtained from sequencing rRNA 16s amplicon. Taxon data were converted to genus since not all works present results in species resolution.

We downloaded data from 44 bladder cancer samples (PRJNA185252) and searched for nonhuman sequences aiming to obtain taxonomic information from the bladder cancer microbiome. After quality filtering and human sequence removal, an average of 180 thousand reads per sample were obtained. The most abundant bacterial genera found in bladder cancer tissues are demonstrated in Figure 1A and Supplementary Table 4. Since there is scarce information about bladder cancer metagenomics, urine metagenomic data were also included (Figure 1B) [56–60].

Despite finding some common genera, the hypergeometric enrichment test did not indicate any significant overlap between the analyzed data (p-adj > 0.05; Supplementary Table 5). However, two genera were present in all six studies: Finegoldia and Streptococcus. Data from Bučević Popović et al. (2018) were the only data that presented detailed taxonomic information from every studied case, and majority of the genera were also found by the WGScd approach (Figure 1B).

**Figure 2:**
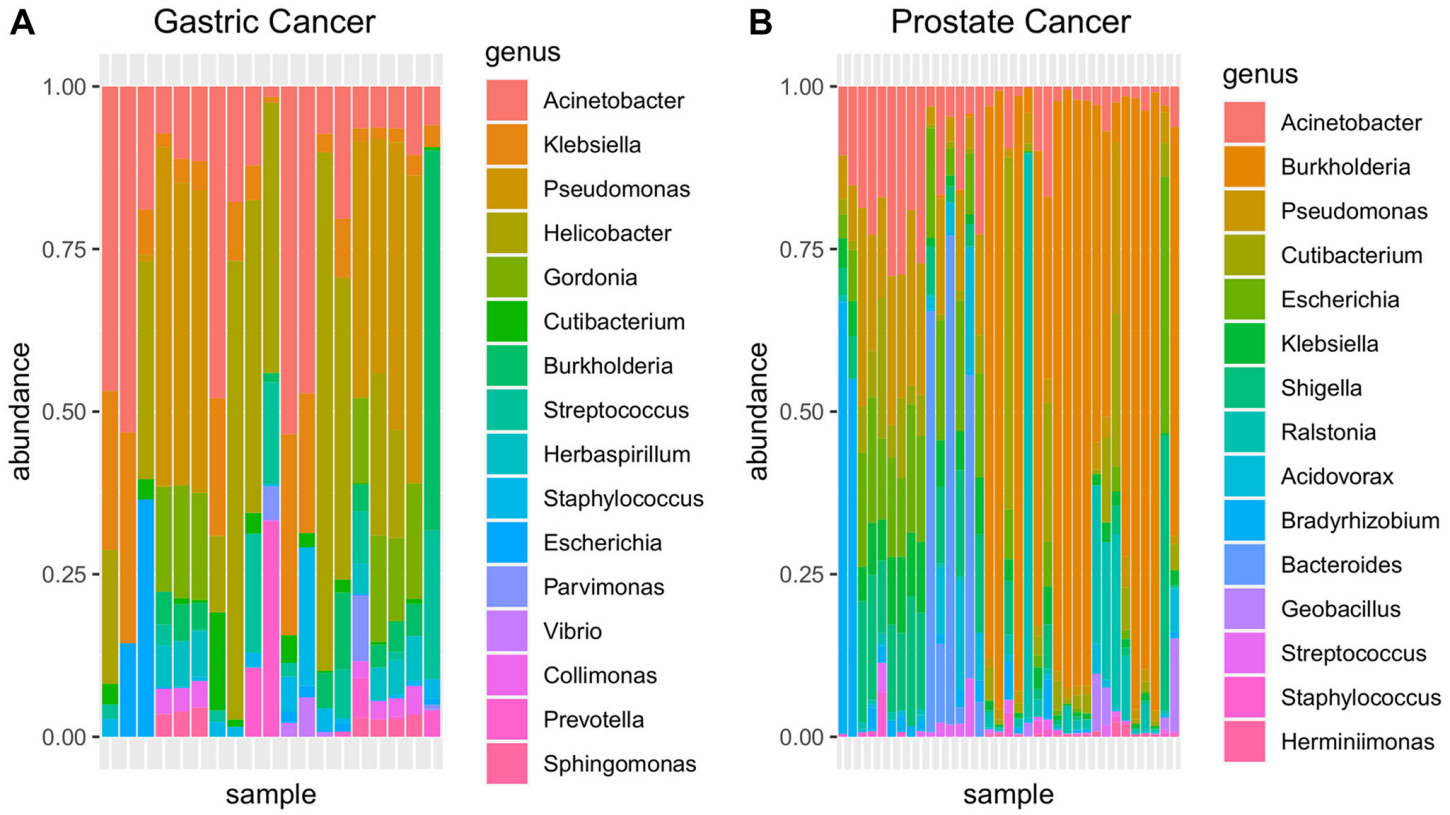
Metagenomic relative abundance in the genus rank from genomic sequencing of (**A**) gastric and (**B**) prostate cancers (WGScd).

The metagenomic data obtained from genomic experiments from prostate and gastric cancers are shown in Figure 2. Rarefaction curves indicate that in most samples, the employed strategy generated sufficient data to represent the bacterial diversity of each sample (Supplementary Figure 1).

**Figure 3:**
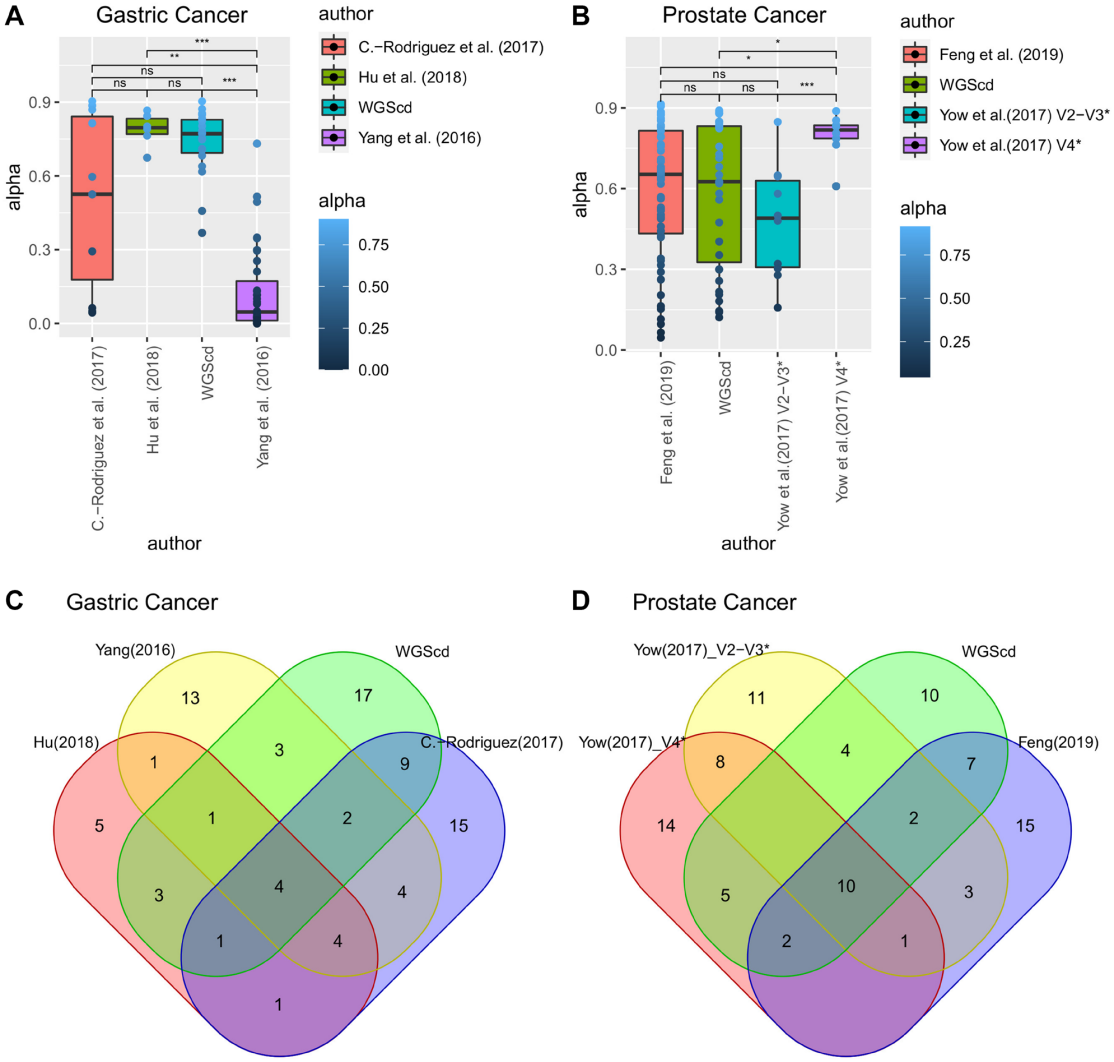
In (**A**) and (**B**), alpha diversity box plot: comparison with literature data indicates that metagenomic analyses from WGScd seem to be as capable of representing community diversity as are dedicated metagenomic analyses. In (**C**) and (**D**), presence/absence Venn diagram: Gastric and prostate cancers microbiome profile obtained from WGScd compared with dedicated metagenomic data of gastric and prostate cancers. Taxon data were converted to genera since not all works present results in species resolution. *Yow et al. (2017) performed two different analyses: v2/v3 rRNA 16s regions and v4 rRNA 16s region.

To compare the WGScd metagenomic findings with data from exclusively metagenomics studies [61–65], we first filtered out low abundance taxa of all samples (read counts < 10) and compared alpha diversity indices among samples with those found in other studies with same cancer types. The results showed that WGScd metagenomic analysis had alpha diversity values similar to those obtained from metagenomic sequencing (Kruskal–Wallis and Dunn’s post hoc test; adjusted *p* value > 0.05; Supplementary Table 6; Figure 3A and 3B).

After correcting for multiple testing, the hypergeometric enrichment test did not indicate any significant overlap among cancer metagenomic results (p-adj > 0.05; Supplementary Table 7). In both cancer types, we found several genera present in WGScd analyses and all metagenomic experiments: four in gastric cancer (Helicobacter, Neisseria, Prevotella, and Streptococcus) and ten in prostate cancer (Escherichia, Pseudomonas, Ralstonia, Acinetobacter, Corynebacterium, Rhodococcus, Staphylococcus, Sphingomonas, Streptococcus, and Acidovorax) (Figure 3C and 3D).

### sncRNAs analysis

**Figure 4:**
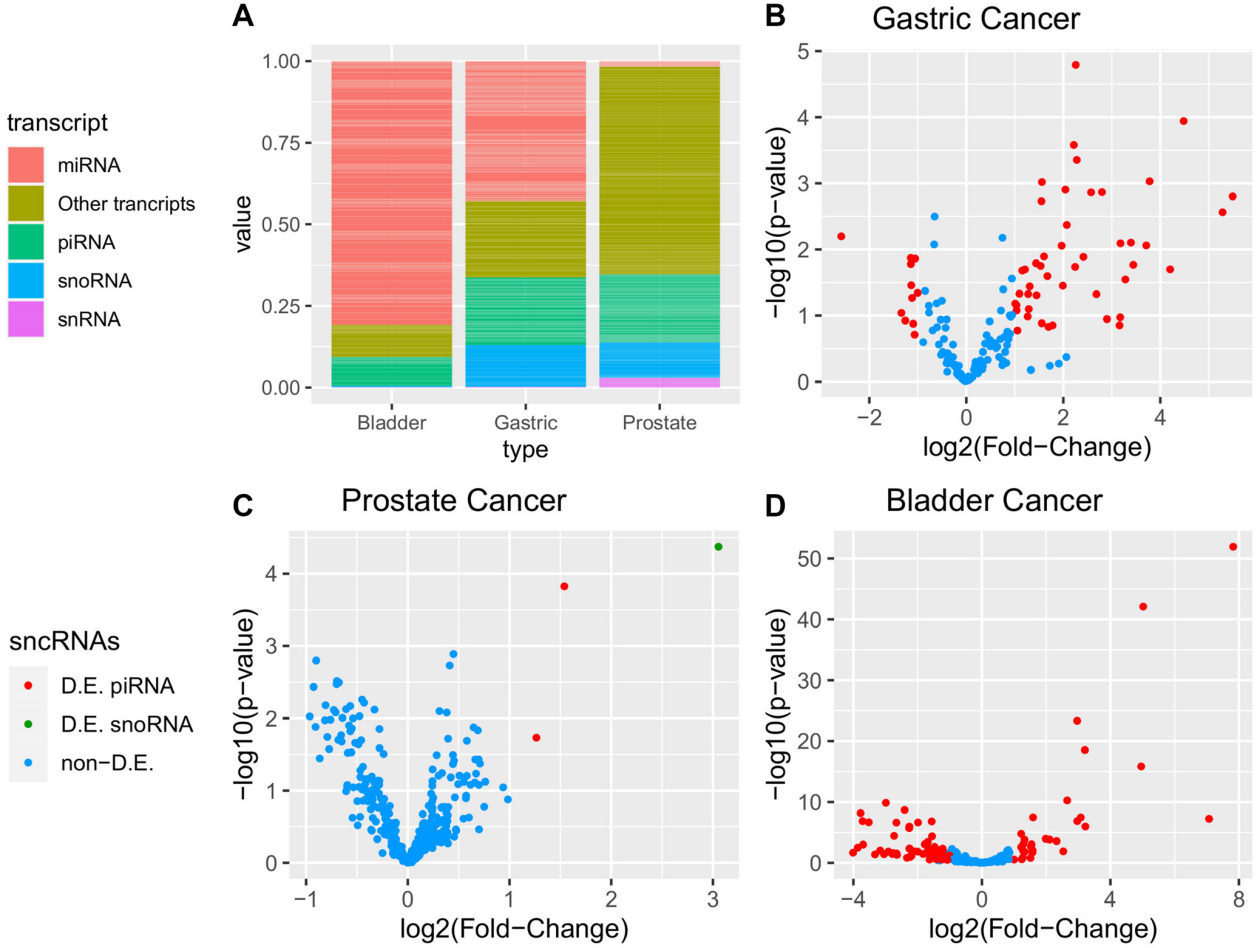
Additional sncRNAs differential expression analysis of gastric, prostate, and bladder cancers obtained from miRNAs expression analyses data. In (**A**), sncRNAs relative abundance of each sequencing. In (**B**), (**C**) and (**D**) volcano plot identifying differentially expressed (DE) sncRNAs (adjusted *p*-value < 0.05; |log2(fold-change)| > 1).

For bladder cancer, we analyzed an average of 16 million (Mi) known transcript reads per sample, including ~81% miRNA reads, ~9% piRNA, and ~10% other transcripts. There was an average of 0.8 Mi reads per sample in gastric cancer, of which ~43% were miRNA reads, ~22% were piRNA reads, 13% were small nucleolar RNA (snoRNA) reads, and ~22% were other transcripts. For prostate cancer peripheral blood, we quantified 1.3 Mi reads per sample, from which ~21% were piRNA reads, ~11% were snoRNA reads, ~3% were snRNAs, ~2% were miRNA reads, and ~63% were other transcripts (Figure 4A).

Comparing gastric cancer sncRNA expression with that of noncancer gastric samples, we identified 57 DE piRNAs, of which 46 were upregulated and 11 were downregulated (Figure 4B). Regarding sncRNA expression in prostate cancer peripheral blood samples with that of noncancer patients, we were able to identify two upregulated piRNAs and one upregulated snoRNA (Figure 4C). Comparing bladder cancer sncRNA expression with noncancer bladder samples, we identified 102 DE piRNAs, of which 29 were upregulated and 73 were downregulated (Figure 4D). Supplementary Table 8 contains the list of DE sncRNAs.

### Analyzing common variations in tumor and adjacent-to-tumor samples

After filtering, 7,443 somatic variants in tumor samples and 7,469 variants in adjacent samples were identified. Comparing tumors with adjacent to tumor samples, we found 1,635 common variants in 1,084 genomic positions, most of which were single nucleotide variants (1,021; 94.9%). Small deletions (30; 2.8%) and small insertions (25; 2.3%) were also found. Most variants had been previously reported (1,036; 95.6%), and 48 (4.4%) were unreported mutations.

**Figure 5:**
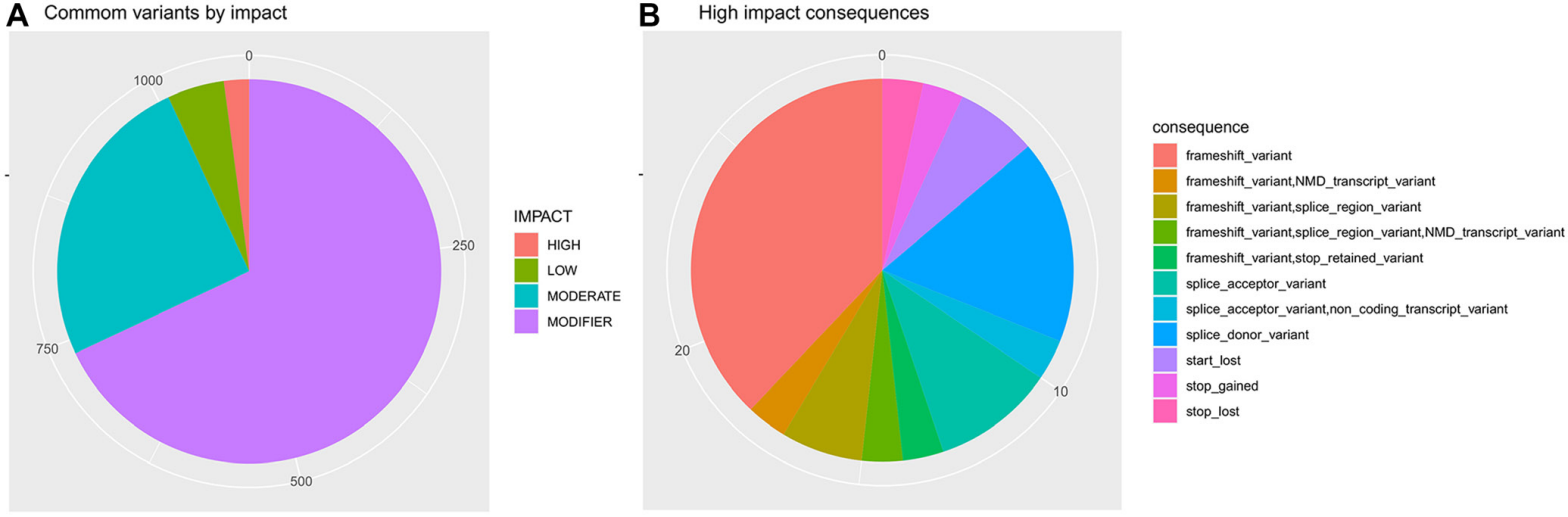
Somatic variants identified in both tumor and adjacent tissue. In (**A**) common variants by impact. In (**B**) potential consequences from high impact common variants. Variants impact and consequences were predicted by the ENSEMBL VEP (https://www.ensembl.org/info/genome/variation/prediction/predicted_data.html).

This analysis was able to identify 23 high-impact common variants (Supplementary Table 9; Figure 5), including 11 frameshifts, two start-loss variants, one stop-loss variant, and one stop-gained variant. From total, 144 common variations were previously reported for gastric cancer on the COSMIC cancer dataset (https://cancer.sanger.ac.uk/cosmic) [66].

## DISCUSSION

Obtaining financial support for cancer research remains a challenge. Additionally, recruiting patients for such investigations also represents a critical step. Therefore, optimizing cancer research by reducing the number of expensive rounds of sequencing experiments and exploring the produced data by additional innovative approaches represents an option to overcome the limitations of collecting human biological samples and the scarce availability of resources to cover the costs of high-throughput experiments. Thus, three different approaches are proposed to better explore NGS data: (i) obtaining metagenomic data from genomic sequencing; (ii) capturing additional sncRNAs from miRNA sequencing; and (iii) analyzing common variants in tumor and adjacent to tumor samples.

Metagenomics has become a new player in cancer research that has reached clinical practice [67]. Although it has been explored in many types of tumors, the role of the microbiome in bladder cancer remains obscure. Bladder epithelium and urine have been considered sterile in healthy individuals; however, new evidence recently demonstrated that the urinary tract also harbors a specific microbiome [56] that may participate in many diseases, including cancer.

Our results showed that metagenomic analyses using genomic data are viable. However, several discussions may emerge from this approach regarding data reliability, such as whether contamination may mask results and whether combined acquisition of human and nonhuman sequences produces the same results as those from solely human or solely nonhuman experiments.

Regarding contamination, both metagenomic and WGScd analyses share similar vulnerabilities. Tissue collection, laboratory handling, and even sterile reagents are not free of contaminants, since sequencing does not require exclusively viable microorganisms, and fragments of inert DNA may be sequenced and included in downstream investigations. Finding such contaminants remains a challenge, even for metagenomics experiments. Filtering contaminants should not be disregarded, both during classical metagenomics experiments and WGScd, and a critical assessment of results is essential for any potential clinical application. Nevertheless, as demonstrated, a significant part of the bacterial presence in NGS data [68–71] is obtained from sequenced tissue and potentially represents tissue microbiome.

A comparison of WGScd results with metagenomics experimental results strongly suggested similarities between the two methods. According to these preliminary results, it seems viable to capture metagenomics information and save time, lab work, and financial resources by looking at already produced data from genomic sequences.

Another question arises from the applicability of WGScd for other tumor sites. To shed light on this question, additional analyses were carried out, and gastric and prostate cancer data were also tested. Compared with other reports, our results seem to be as reliable as those from metagenomic investigations.

An additional application of the proposed strategy is the investigation of rare tumors and sites with complex accessibility, such as the brain. These data could be reinvestigated from a metagenomic standpoint to provide insights on potential interactions that could be addressed both for carcinogenic understanding and clinical applications.

Experiments should be designed to search for both types of data and perform integrative investigation of genomics and metagenomics from the same samples. In addition to conserving time, saving human and financial resources, and reducing the number of recruited patients, as discussed, this integrative analysis provides an additional advantage of joining genomics and metagenomics from the same clinical situation instead of analyzing each from a different set of patients.

Another approach for better exploring NGS data is simultaneous analysis of diverse sncRNAs. A significant number of miRNA NGS experiments are currently reported due to their potential role as biomarkers. They are essential for cellular and tissue homeostasis and are involved in posttranscriptional gene regulation [18]. Sequencing is relatively inexpensive, and the results for cancer research are relevant. However, other promising sncRNAs are involved in critical biological processes, such as piRNAs [72], and could be analyzed from the same raw dataset.

By exploring this possibility in three cancer types, a large number of additional ncRNAs were identified and quantified, confirming our hypothesis. This relevant information adds strength and value to such analyses, introducing new players and enabling an integrative interpretation of the role of these sncRNAs in cancers and other biological processes.

However, it should be noted that library preparation has an essential role in ncRNA identification [73]. Some sncRNA sequencing requires size selection of larger RNA fragments when compared to exclusive miRNA sequencing. Library preparation from each analyzed dataset had different sizes, namely, 18–30 nucleotides (nts) for bladder tissue, 10–40 nts for prostate tissue, and 15–35 nts for gastric tissue. Larger size selections allowed for identification of a greater variety of sncRNAs, although with lower expression levels. Conversely, smaller size selection resulted in a lower variety of molecules but higher expression levels. This should be taken into consideration for experimental design.

Finally, RNA-seq data were analyzed to find common variants for both tumor and adjacent to tumor tissue in gastric cancer. Using RNA-seq data to identify genomic variants is challenging given the technical computational limitations due to intrinsic complexity of transcriptome, which increases the rate of false-positives compared to DNA-seq data [74, 75]. Alignment of RNA-seq data is more complex than that of DNA-seq data because in mRNA, introns are removed by splicing, which in turn could be identified as deletions. Similarly, RNA editing and polyadenylation processes introduce additional mismatches not found in usual DNA-seq alignment [75]. The false-positives introduced by RNA editing can be minimized since most RNA editing sites are already described [76] and variations in these genomic positions can be removed.

In this particular analysis, removing RNA editing variants was not considered since we aimed to find common variants from tissue adjacent RNA-seq data and tumor DNA-seq data. Somatic variants in both tissues at an RNA edit position are more likely to be true genomic variants than false-positive findings.

Another putative limitation is calling only mutations in expressed transcript, leaving out both unexpressed genes and intronic/intergenic regions. However, using RNA-seq data to call variants allows for the identification of tissue-specific variant expressions, which are relevant for translational approaches.

Our results identified several common deleterious variants in both tissues. Although these findings may need further experimental investigation, based on the field cancerization hypothesis, this approach may shed light on early steps of carcinogenesis. The proof of concept is again strengthened, especially when regarding the strategy of obtaining as much data as possible from each experiment, allowing more comprehensive interpretations and optimizing resources.

Altogether, our results strengthen the hypothesis that abundant additional and potentially useful information can be extracted from NGS. Moreover, the integrated investigation of every available information should provide a broader and more robust interpretation of the molecular scenario from each experiment.

## SUPPLEMENTARY MATERIALS






