# Supplementary material for: Treasures from trash in cancer research
Source: Oncotarget. 2022 Nov 17;13:1246–57. doi: 10.18632/oncotarget.28308 (PMC9671455; doi:10.18632/oncotarget.28308)
Supplement: Supplementary file 1 [file oncotarget-13-28308-s001.pdf]

Treasures from trash in cancer research

SUPPLEMENTARY MATERIALS

Command lines for each pipeline

Metagenomic analysis from genomic data

```
# quality control
trimmomatic-0.36.jar PE -threads 30 -phred33 \
[input_file.fq] [output_file.fq] \
ILLUMINACLIP:TruSeq3-SE.fa:2:30:10 \
LEADING:10 TRAILING:10 SLIDINGWINDOW:3:15 \
MINLEN:80

# mapping and quantification
centrifuge -p 25 $path_database/p+h+v \
-1 ID_1.fastq -2 ID_2.fastq -U ID.fastq \
--report-file ID_rep.txt -S ID_res.txt

#recentrifuge
python3.6 $Path_recentrifuge/rcf  -n $Path_recentrifuge/taxdump\
-y 50 -f ID_res.txt
```

sncRNAs simultaneous analyses

```
# quality control
trimmomatic-0.36.jar SE -threads 30 -phred33  \
[input_file.fq] [output_file.fq] \
ILLUMINACLIP:TruSeq3-SE.fa:2:30:10 \
LEADING:10 TRAILING:10 SLIDINGWINDOW:3:20 \
MINLEN:17

# mapping sorting and indexing
STAR  --runThreadN 30 --genomeDir hg19/star_genome \
--readFilesIn [output_file.fq] --outReadsUnmapped Fastx  \
    --outFilterMultimapNmax 100 --outFileNamePrefix [prefix] \
     --outFilterMismatchNmax 2   --outFilterMatchNmin 16     \
     --outFilterScoreMinOverLread 0 --outFilterMatchNminOverLread 0

# quantifying
htseq-count --type=miRNA --stranded=yes --idattr=Name \
<file.sam> miRbase_annotation/hsa_v20.gff     \
    --samout <output.sam> > mirna.count.txt

# to quantify other sncRNAs, the annotation was changed
```

Analyzing common variations in tumor and adjacent samples

```
# quality control
trimmomatic-0.36.jar PE -threads 30 -phred33 \
[input_file_R1.fq] [input_file_R2.fq] \
[output_file_R1.fq] [output_file_R2.fq] \
```

```
[unpaired_R1.fq] [unpaired_R2.fq]                         \
ILLUMINACLIP:TruSeq3-PE.fa:2:25:10 LEADING:10              \
TRAILING:10 SLIDINGWINDOW:5:25 MINLEN:50

# mapping DNA samples
bwa mem -t 30 hg19_v37.fasta                              \
[input_file_R1.fq] [input_file_R2.fq] > [output.sam]


# mapping RNA samples
STAR --runThreadN 30 --genomeDir hg19/star_genome                          \
--readFilesIn  [input_file_R1.fq] [input_file_R2.fq]        \
--outFileNamePrefix [prefix] --sjdbGTFfile hg19.annotation.gtf


# Sorting, removing duplicates and indexing for all samples
picard SortSam [input.sam] O=[output.bam] SORT_ORDER=coordinate

picard CollectAlignmentSummaryMetrics R=hg19_v37.fasta        \ I=[input.bam] O=alignment_metrics.txt
picard MarkDuplicates I=[input.bam] O=[output.bam]                \METRICS_FILE=alignment_metrics.txtREMOVE_
DUPLICATES=TRUE
picard BuildBamIndex INPUT=[output.bam]


# Base recalibration for all samples
gatk BaseRecalibrator -I [input.bam] -R hg19_v37.fasta  \
--known-sites dbsnp_150.hg19.vcf.gz -O recal_data.table
gatk ApplyBQSR -R hg19_v37.fasta -I [input.bam]                     \
--bqsr-recal-file recal_data.table -O [output.bam]

# Base calling and filtering out low coverage variants
bcftools mpileup -f hg19_v37.fasta -b list_of_samples.txt   \
--thread 30 -R exome_regions.bed.gz |                              \
bcftools call -mv -O z -o [output.vcf.gz]
bcftools view
-i '(DP4[2]+DP4[3])>5 && ((DP4[2]+DP4[3])/sum(DP4)>0.2)' [input.vcf.gz] -O z -o output.vcf.gz

# Filtering out blood (germlines) variants
bcftools isec -C -w1 [input.vcf.gz] [blood.vcf.gz] \
-O z -o [somatic.vcf.gz]

# Identifying Common variants
bcftools isec -w1 -n=2 [tumor.vcf.gz] [adjacent.vcf.gz] \
-O z -o [output.vcf.gz]
```

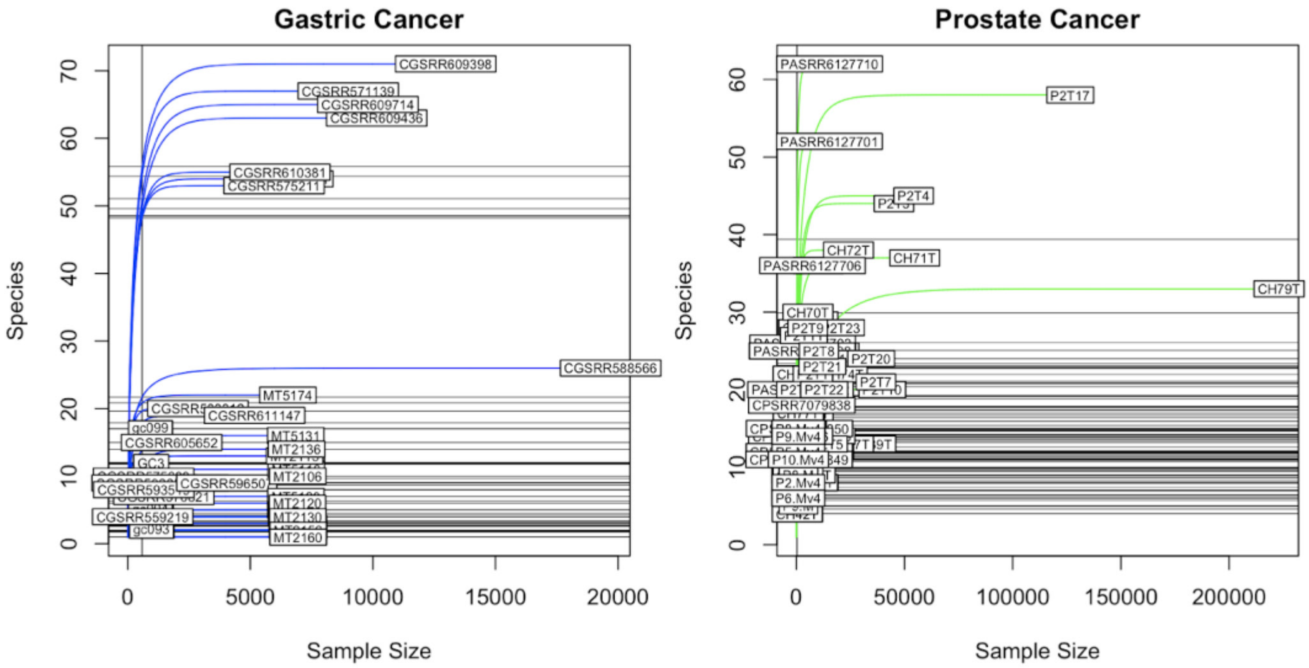

Supplementary Figure 1: Gastric cancer and prostate cancer rarefaction curves.

Supplementary Table 1: Twenty gastric cancer patients and four samples per patient obtained from SRA (https://www.ncbi.nlm.nih.gov/sra)

	DNA		RNA	
**Patient**	**Blood**	**Tumor**	**Adjacent**	**Tumor**
1	SRR8244841	SRR8244890	SRR8281317	SRR8281397
3	SRR8244839	SRR8244892	SRR8281319	SRR8281399
5	SRR8244837	SRR8244886	SRR8281321	SRR8281401
6	SRR8244838	SRR8244885	SRR8281322	SRR8281402
8	SRR8244836	SRR8244887	SRR8281324	SRR8281404
12	SRR8244914	SRR8244922	SRR8281328	SRR8281408
15	SRR8244909	SRR8244917	SRR8281331	SRR8281411
16	SRR8244910	SRR8244918	SRR8281332	SRR8281412
17	SRR8244907	SRR8244915	SRR8281333	SRR8281413
19	SRR8244905	SRR8244923	SRR8281335	SRR8281415
20	SRR8244906	SRR8244924	SRR8281336	SRR8281416
21	SRR8244852	SRR8244810	SRR8281337	SRR8281417
22	SRR8244851	SRR8244809	SRR8281338	SRR8281418
23	SRR8244850	SRR8244812	SRR8281339	SRR8281419
24	SRR8244849	SRR8244811	SRR8281340	SRR8281420
25	SRR8244848	SRR8244806	SRR8281341	SRR8281421
26	SRR8244847	SRR8244805	SRR8281342	SRR8281422
29	SRR8244854	SRR8244814	SRR8281345	SRR8281425
30	SRR8244853	SRR8244813	SRR8281346	SRR8281426
32	SRR8244866	SRR8244792	SRR8281348	SRR8281428

Supplementary Table 2: Trimmomatic parameters settings for each analysis

Parameter	WGScd	sncRNAs	RNA-seq variant call	Exome variant call
**Paired**	PE	SE	PE	PE
**Quality Value (QV)**	15	20	25	25
**Sliding Window size**	3	3	5	5
**Minimum read length**	80	17	50	50

Supplementary Table 3: Relative abundance of the top 40 genera from metagenomic experiments

Author	Abundance	Cancer type
**Castaño-Rodriguez et al. (2017)**	0.943	Gastric
**Feng et al. (2019)**	0.957	Prostate
**Hu et al. (2018)**	0.963	Gastric
**WGScd**	0.987	Gastric / Prostate
**Yang et al. (2016)**	0.995	Gastric
**Yow et al. (2017) V2-V3**	0.985	Prostate
**Yow et al. (2017) V4**	0.990	Prostate

Supplementary Table 4: All bacteria found in the bladder samples (species, genus and read count). See Supplementary Table 4

Supplementary Table 5: Hypergeometric enrichment test with adjusted *p*-values from overlap comparison of genus identified in bladder cancer samples with Whole Genomic Sequencing captured data (WGScd) approach and metagenomics experiments

Bladder Cancer	Wu et al. (2018)	Mai et al. (2019)	Popovic et al. (2018)	WGScd
Xu et al. (2014)	0.824	0.22	1	1
Wu et al. (2018)	–	0.824	1	1
Mai et al. (2019)	–	–	1	1
Popovic et al. (2018)	–	–	–	1

Supplementary Table 6: Gastric cancer and prostate cancer alpha diversity comparison between WGScd and metagenomics experiments

	Gastric				Prostate		
	**Feng et al.**	**WGScd**	**Yow_V4**		**C. Rodrig**	**Hu et al.**	**WGScd**
**WGScd**	–0.026025<br>0.4896			**Hu et al.**	–1.248440<br>0.2006		
**Yow_V4**	1.491526<br>0.1902	1.438001<br>0.1448		**WGScd**	–1.260627<br>0.2800	0.333117<br>0.3695	
**Yow_V2V3**	–2.383412<br>0.0422	–2.257650<br>0.0471	–2.963114<br>0.0091*	**Yand et al.**	3.365194<br>0.0015*	4.066512<br>0.0001*	5.758051<br>0.0000*

*Significant differences. Kruskall-Wallis test followed by Dunn's post hoc test, where the upper number for each comparison is Dunn's pairwise z test statistic and the lower number is the *p* value associated with the test.

Supplementary Table 7: Hypergeometric enrichment test with adjusted *p* values from overlap comparison of genera identified in gastric and prostate cancers with WGScd approach and other metagenomics experiments

Gastric Cancer	Yang (2016)	WGScd	Castaño-Rodriguez (2017)
**Hu (2018)**	1	1	1
**Yang (2016)**	–	1	1
**WGScd**	–	–	1
**Prostate Cancer**	**Yow(2017)_V2–V3**	**WGScd**	**Feng (2019)**
**Yow (2017)_V4**	0.119	0.532	0.631
**Yow (2017)_V2-V3**	–	0.593	0.59
**WGScd**	–	–	0.033

Supplementary Table 8: Complete list of DE sncRNAs found in all three analyses, with piRNAs indicated with (*) indicating overlapping piRNA sequences merged with BEDtools (version 2.17; https://bedtools.readthedocs.io), to avoid ambiguous recognition. See Supplementary Table 8

Supplementary Table 9: High impact somatic variants identified in both tumor and adjacent to tumor tissue from gastric cancer patients

Location	Variant	Gene	Reference SNP ID
11:74419346-74419346	T	*CHRDL2*	–
12:120961807-120961807	C	*COQ5*	rs1259777611
13:52718051-52718051	T	*NEK3*	–
13:52718057-52718057	T	*NEK3*	COSV51618297
14:21458320-21458324	–	*METTL17*	rs36002749
16:31770696-31770696	A	*ZNF720*	rs34487972
16:31985158-31985158	C	*RP11-170L3.7*	–
16:57095842-57095846	–	*NLRC5*	rs71383216
17:34522695-34522695	G	*CCL3L3*	rs201343647, COSV65674832
17:43192550-43192550	C	*PLCD3*	rs35911033, COSV59558342
17:7290695-7290695	G	*TNK1*	rs7220814
2:191184475-191184475	G	*HIBCH*	rs291466, CM1311889
2:238244777-238244777	A	*COL6A3*	rs995147980
20:44528474-44528474	G	*PLTP*	rs113023458
21:28216059-28216059	G	*ADAMTS1*	rs400852
21:46924424-46924434	–	*COL18A1*	rs1555874527
22:50705304-50705304	T	*MAPK11*	rs760747
3:108634973-108634973	A	*GUCA1C*	rs10933973
3:73111481-73111481	A	*EBLN2*	rs3832186
3:73111482-73111482	A	*EBLN2*	rs3832186, COSV55593932
7:76071184-76071184	G	*ZP3*	–
9:43844264-43844265	–	*CNTNAP3B*	rs200487787, COSV59543099, COSV59548452
X:19375782-19375782	C	*PDHA1*	rs2229137, CM119793, CM950922, COSV58826890
